# A renal angiomyolipoma with a challenging presentation: a case report

**DOI:** 10.1186/s13256-021-03073-0

**Published:** 2021-09-27

**Authors:** H. Bouaziz, M. Ghalleb, N. Tounsi, N. Riahi, H. Bouzaiene, J. Ziadi, J. Ben Hassouna, M. Slimane, K. Rahal

**Affiliations:** 1 University of Tunis ElManar, Tunis, Tunisia; 2 Department of Surgical Oncology, Salah Azaiez Institute, 1062 Tunis, Tunisia; 3 Department of Cardiovascular Surgery, La Rabta University Hospital, 1062 Tunis, Tunisia

**Keywords:** Angiolipoma, Venous thrombosis, Nephrectomy, Cardiac surgery

## Abstract

**Introduction:**

Renal angiomyolipoma is considered a benign mesenchymal tumor composed of fat, smooth muscle, and blood vessels. It represents 1–3% of solid renal tumors. Despite this tumor’s benignity, it can be aggressive with a locoregional extension.

**Case report:**

A 41-year-old north African caucasian woman consulted with chief complaints of right lower back pain with no hematuria and no urinal sign. Thoracic-abdominopelvic contrast-enhanced computed tomography showed a right inferior polar heterogeneous renal mass complicated with venous thrombus ascending to the right atrium level. The patient underwent radical nephrectomy under extracorporeal circulation and direct supervision of the fatty thrombus at the right atrium level. The postoperative period was uneventful. The final histologic examination was concordant with renal angiomyolipoma.

**Conclusion:**

Renal angiomyolipoma is the most common benign kidney tumor. Despite its benignity, it can be associated with lethal complications such as hemorrhage, and it can also show signs of local extension mimicking malignant tumors. The cornerstone of the treatment remains surgery.

## Introduction

Renal angiomyolipoma (AML) is a benign mesenchymal tumor composed of fat, smooth muscle, and blood vessels. It represents 1–3% of solid renal tumors.

AML is the most common type among benign kidney tumors. It accounts for 0.3–3% of all renal masses [1].

Approximately 80% of these tumors are sporadic. However, in some cases, they are associated with tuberous sclerosis complex [1].

Despite this tumor’s benignity, it can be aggressive with a locoregional and venous extension.

We report a case of renal angiomyolipoma mimicking kidney cancer with a thrombotic extension to the renal vein and the inferior vena cava, which required extracorporeal circulation preoperatively.

## Case presentation

A 41-year-old north African caucasian woman, who had no personal or familial history, consulted with the chief complaints of right lower back pain with no hematuria and no urological signs.

On examination, the abdomen was depressible, with no palpable masses, no lumbar pain, and no palpable lymphadenopathy.

**Fig. 1:**
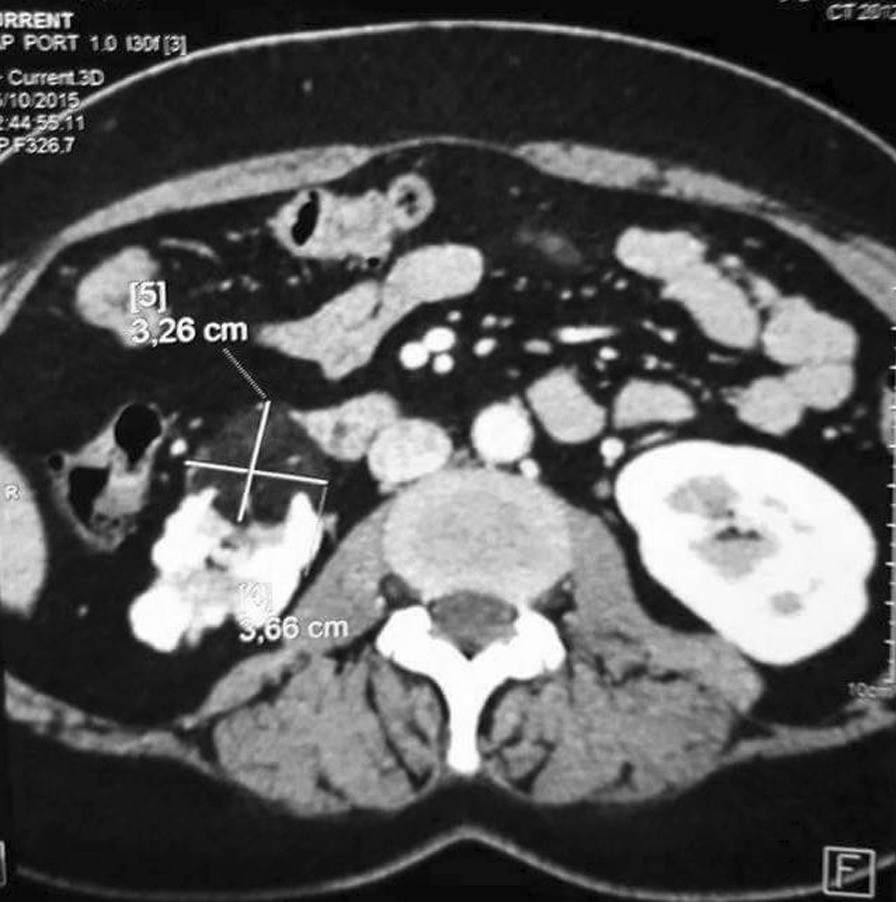
Right inferior polar heterogeneous renal mass with a predominant fatty component

**Fig. 2:**
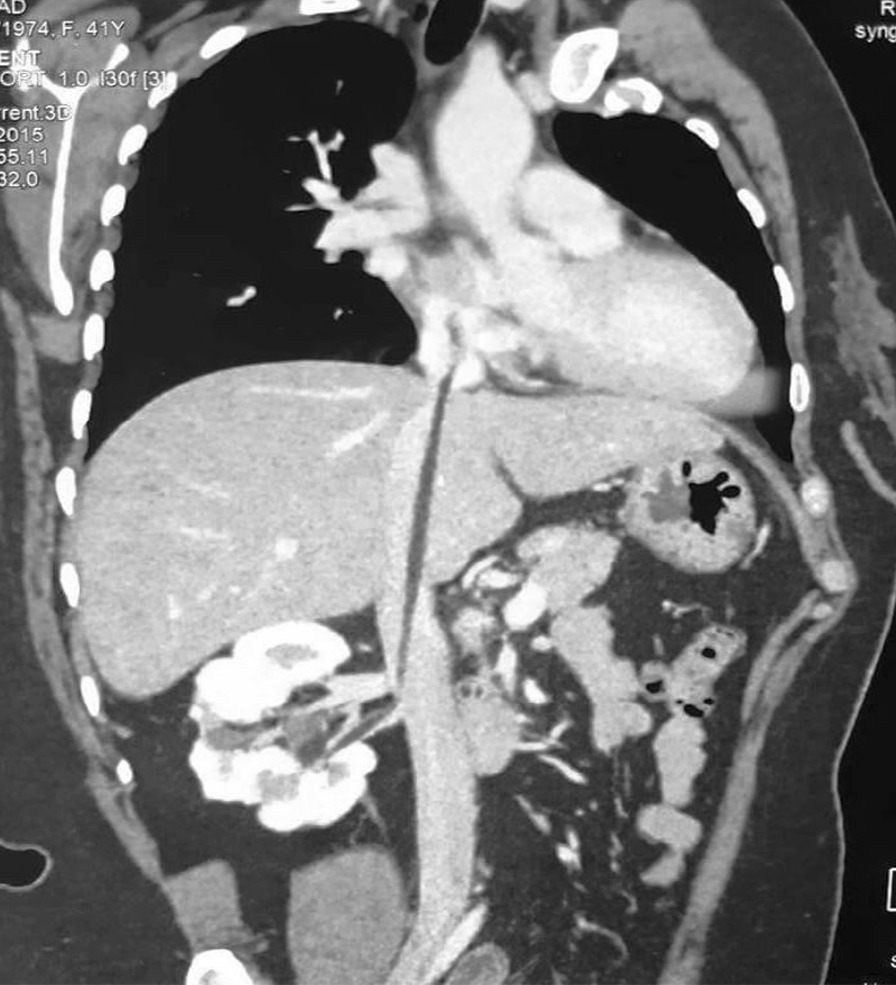
Computed tomography scan showing renal angiomyolippma with inferior vena cava thrombus. The thrombus extends up to the right atrium

**Fig. 3:**
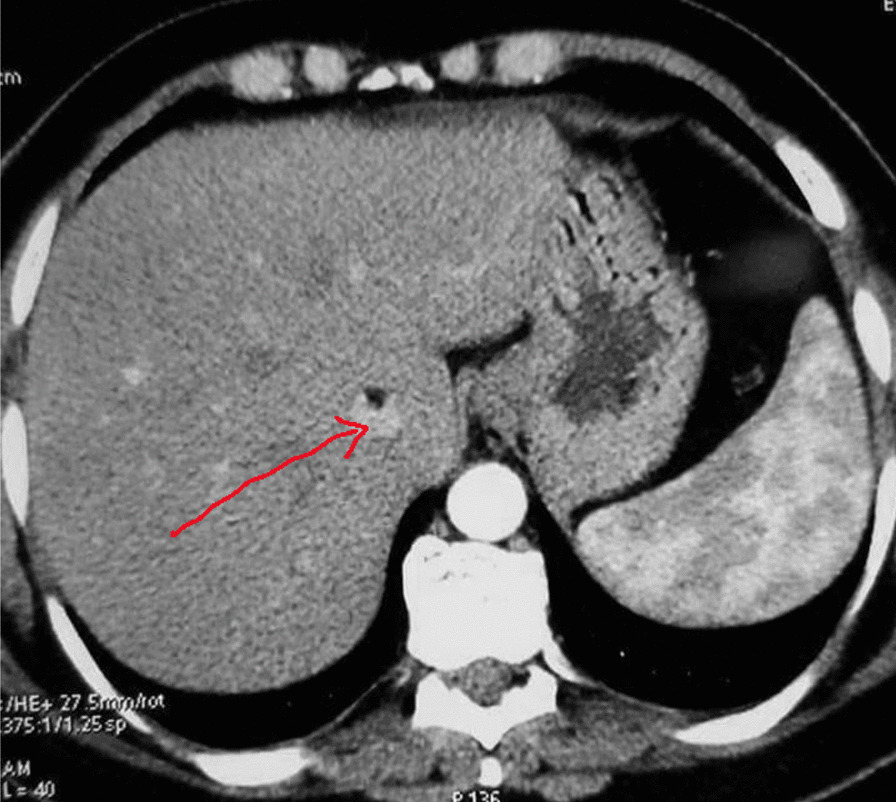
Computed tomography scan showing thrombus in the vena cava (arrow)

Biochemistry analysis was regular. Abdominal ultrasound showed a suspicious kidney mass. Thoracic-abdominopelvic contrast-enhanced computed tomography (CECT) showed a right inferior polar heterogeneous renal mass with a predominant fatty component and exophytic development (Figure 1). It also showed the presence of a tumoral thrombus going from the renal vein until the end of the inferior vena cava, with no calcifications, intratumoral necrosis, or intratumoral aneurysm. (Figures 2, 3). CECT urogram showed that this mass measured 4 × 1.2 cm with extension to the right renal vein and the inferior vena cava to the right atrium.

The contralateral kidney was normal.

## Treatment and outcome

The decision in multidisciplinary consultation was to perform a radical nephrectomy with thrombus extraction. Due to the unusual extension of the tumor and the risk that a part of the thrombus may detach and cause pulmonary embolism, the decision was rapid surgical intervention.

We first performed an exploratory laparotomy. We found a 6 cm inferior polar tumor; it had a greasy consistency and thrombosed the inferior vena cava. The right colon was reflected, allowing access to the retroperitoneal space and dissection of the whole right kidney. We started with ligation of the right renal artery, and the kidney was left attached only by the renal vein with the thrombus inside.

**Fig. 4:**
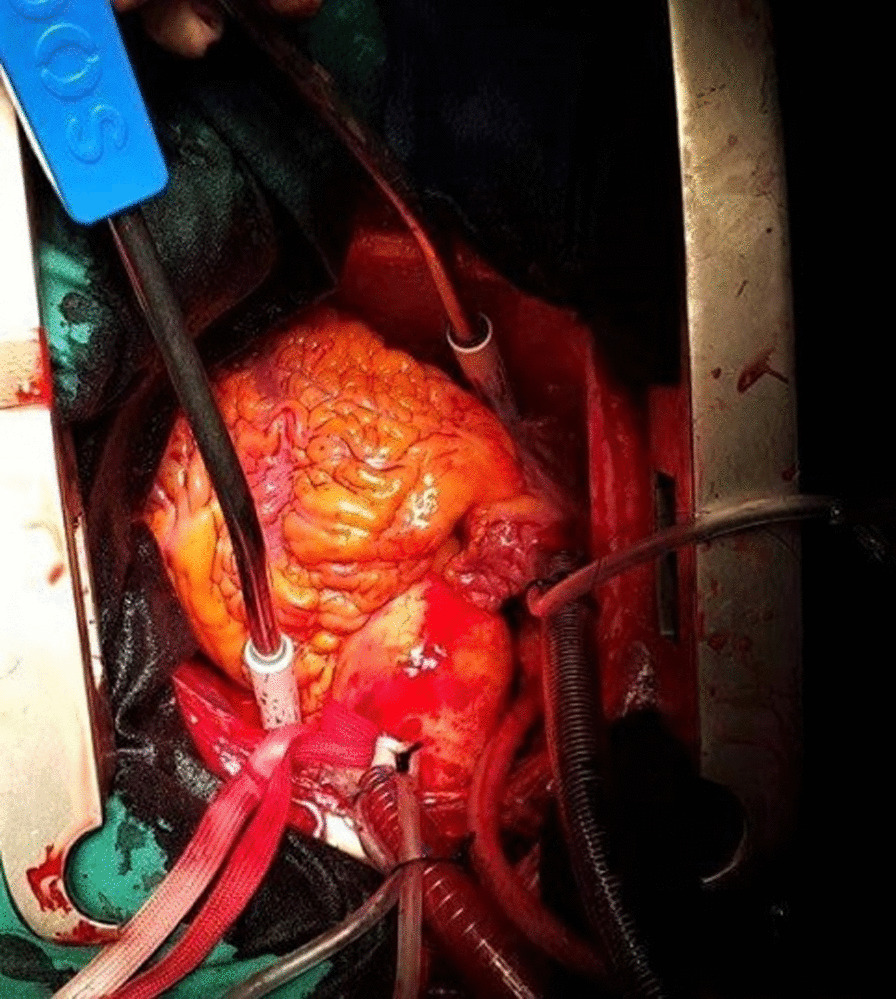
Extracorporeal circulation for thrombus extension into the heart

We secondarily performed median sternotomy, and opened the pericardium. The right atrium, aorta, intrapericardial inferior vena cava, and right superior pulmonary vein were exposed. The patient was put on extracorporeal circulation. Ascending aorta and right atrium were cannulated. A vent cannula was inserted in ascending aorta (Figure 4). Aorta was clamped. When the right atrium was opened, the distal part of the thrombus was visualized.

**Fig. 5:**
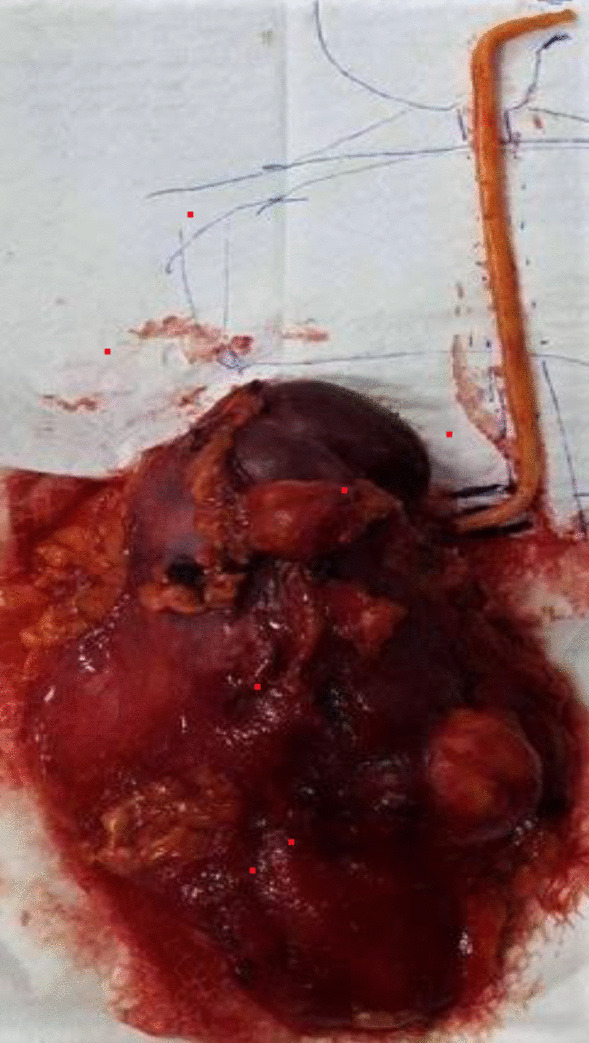
Picture of the operative specimen showing the renal angiomyolippma with the fatty thrombus

A circumferential incision of the renal vein was done, and the kidney was extracted along with the fatty thrombus attached (Figure 5) under visual control from the right atrium.

The postoperative course was unremarkable; the patient was extubated 24 hours after and discharged 5 days after the surgery with a regular biologic analysis.

The final histologic result showed an enlarged kidney measuring 16 × 10 × 4 cm and weighing 300 g; the examination showed a sizeable renal angiomyolipoma that measured 5.5 × 3 cm without any sign of malignancy, with an inferior vena cava thrombus that had the exact characteristics of the renal angiomyolipoma**.**

The patient was last seen at our institution 10 months after surgery. The patient was doing well with no sign of relapse. Based on the benign nature of the tumor, she was referred to her original hospital.

## Discussion

Renal angiomyolipoma is a benign mesenchymal tumor first defined in literature by Fisher in 1911; it represents 1–3% of the solid tumors [1].

The prevalence of AML is 0.28% in males and 0.6% in females [1].

Prasad *et al*. stated that the prevalence of renal angiomyolipoma is higher in middle-aged women [2].

A historical series demonstrates that renal angiomyolipoma affects more women than men, with a sex ratio of 2:1 [1, 3, 4].

A Japanese study that included 17,941 Japanese patients demonstrated that the female-to-male ratio was 2.5, and the mean age of the patients was 55.89 ± 14.49 years.

In literature, the classical triad of symptoms in AML is flank pain, palpable mass, and hematuria [1].

Its main complication is spontaneous hemorrhage in the retroperitoneum or the kidney collecting system, leading to a life-threatening situation [5].

Yiu *et al*. demonstrated in their review of 16 studies that there is no association between the tumor’s size and its aggressive behavior [5].

Tan *et al*. deduced after examining 35 cases that the transformation into a malignant form is more likely when the tumor has an aggressive behavior [6].

The extension of a tumor into the inferior vena cava is commonly seen in renal cell carcinoma but can also rarely be seen in angiomyolipomas, depending on the tumor’s dimensions. The first case of renal angiomyolipoma with an extension to the inferior vena cava was described in 1982 by Kutcher *et al*. [7].

Riviere *et al*. have reported 44 cases of AMLs with renal vein or inferior vena cava (IVC) extension in which almost all the lesions were more prominent than 4 cm [8].

The central location and the localization in the right kidney have been reported as favoring factors for venous invasion. This can be explained by the shorter route to the renal vein [9].

Venous invasion was seen in all types of AML. However, in the reported cases it was mostly associated with epithelioid type AML [10].

In most of the studied cases, the mechanism of invasion was direct invasion by the tumor [8, 10].

The differential diagnoses for macroscopic fat-containing retroperitoneum tumors are AML, lipoma, liposarcoma, teratoma, renal cell carcinoma, and adrenal myelolipoma.

Imaging plays a crucial role in characterizing the tumor as well as revealing its local extent.

On sonography, renal angiomyolipomas are intensely echogenic and may show acoustic shadowing [8].

CT scanning shows good performance in the characterization and diagnosis of angiomyolipoma lesions.

It shows areas of fat attenuation (−10 HU or lower) [9].

In his study, Helenon *et al*. classified the AML as a type 2 tumor. The presence on CT of fat quota into the tumor (≤ −20 UH) and the absence of calcification or necrosis confirm the tumor’s benignity [11].

In 4–5% of AMLs, intralesional fat cannot be detected on CT owing to the small amount of fat within the lesion. These lesions are hyperdense on CT and enhance after contrast administration.

These AMLs represent a diagnostic challenge as they can be mistaken for renal carcinoma.

Magnetic resonance imaging (MRI) does not seem to provide any advantages over CT, except when intravenous contrast administration is contraindicated [12]. The characteristic appearances of angiomyolipomas with MRI include variable areas of high-intensity signal within the tumor on T1-weighted and T2-weighted images.

MRI can also detect fat in these tumors; it can show abnormal tumoral vessels and accurately delineate the local extent [13].

The current management options include observation, embolization, and partial and total nephrectomy.

Oesterling *et al*. recommended that symptomatic tumors less than 4 cm should be observed regularly with CT or ultrasound, whereas those greater than 4 cm should be studied by angiography and considered for arterial embolization or surgery [14].

The tumor’s extension into the renal vein and IVC mandates tumor thrombectomy.

## Conclusion

Our case shows that a renal angiomyolipoma can be aggressive and invade both the right renal vein and the inferior vena cava until the right atrium, necessitating an extracorporeal circulation preoperatively.

## Data Availability

All the data used were taken from the patient’s medical folder available at the archive of our institution.
